# Reviewed article: Santos DS, Viana PVS, Basta PC. Observational study of the coverage rate and survival analysis of COVID-19 vaccination among Indigenous people in Roraima, 2021-2023. Epidemiol Serv Saude. 2025:34;e20240762

**DOI:** 10.1590/S2237-96222025v34e20240762.a

**Published:** 2025-10-24

**Authors:** Patricia Matias Pinheiro

**Affiliations:** 1 Empresa Brasileira de Serviços Hospitalares, Brasília, DF, Brazil

## Peer review A

## Questionnaire

Does the manuscript contain new and significant information to justify publication? Yes

Does the Abstract (Summary) clearly and accurately describe the content of the article? Yes

Is the problem significant and concisely stated? Yes

Are the methods described comprehensively? Yes

Are the interpretations and conclusions justified by the results? Yes

Is adequate reference made to other work in the field? Yes

## Manuscript Structure

Length of article is: Adequate

Number of tables is: Adequate

Number of figures is: Adequate

Please state any conflict(s) of interest that you have in relation to the review of this paper. None

Interest: Good

Quality: Good

Originality: Good

Overall: Good

Recommendation: Minor Revision

## Comments

Descreva com mais detalhes como se dá a coleta e inserção dos dados no sistema utilizado para melhor análise de viés de informação.

15- Discutir a validade externa do estudo e se a amostra é representativa da população indígena brasileira

21- Apresentar a taxa de perdas, para posterior análise dos impactos nos resultados.

